# Modification by hemochromatosis gene polymorphisms of the association between traffic-related air pollution and cognition in older men: a cohort study

**DOI:** 10.1186/1476-069X-12-16

**Published:** 2013-02-15

**Authors:** Melinda C Power, Marc G Weisskopf, Stacey E Alexeeff, Robert O Wright, Brent A Coull, Avron Spiro, Joel Schwartz

**Affiliations:** 1 Department of Epidemiology, Harvard School of Public Health, 677 Huntington Avenue, Boston, MA 02115, USA; 2 Department of Environmental Health, Harvard School of Public Health, 677 Huntington Avenue, Boston, MA 02115, USA; 3 Department of Biostatistics, Harvard School of Public Health, 677 Huntington Avenue, Boston, MA, 02115, USA; 4 Veterans Affairs Boston Healthcare System, 150 S. Huntington Avenue, Boston, MA, 02130, USA; 5 Department of Epidemiology, Boston University School of Public Health, 715 Albany Street, Boston, MA, 02118, USA; 6 Department of Psychiatry, Boston University Medical School, 72 East Concord Street, Boston, MA, 02118, USA

**Keywords:** Aging, Black carbon, Cognitive dysfunction, Epidemiology, Particulate matter, HFE, Hemochromatosis, Gene-environment interaction, Susceptible group

## Abstract

**Background:**

Previous studies found effect modification of associations between traffic-related air pollution and cardiovascular outcomes by polymorphisms in the hemochromatosis gene (HFE). As traffic-related air pollution may impact cognition through effects on cardiovascular health or through mechanisms which may also influence cardiovascular outcomes, we hypothesized that HFE polymorphisms would also modify a previously observed association between traffic-related air pollution exposure and cognition in older men.

**Methods:**

We considered data from 628 participants of the VA Normative Aging Study. We estimated long term exposure to black carbon (BC), a marker of traffic related air pollution, using a spatio-temporal land use regression model. We assessed cognition using the Mini-Mental State Examination (MMSE), a test of global function, and performance on a battery of other tests, covering a wide range of domains. We investigated whether variants of HFE C282Y and H63D modified the association between BC and having a low MMSE score using logistic models with generalized estimating equations and multiplicative interaction terms. Similarly, we assessed whether HFE variants modified the association between BC and performance on the cognitive battery using linear mixed models with multiplicative interaction terms.

**Results:**

Our results suggest modification of the BC-cognition association by HFE C282Y, although the test of interaction did not achieve statistical significance. In multivariable-adjusted models, participants who lacked a HFE C282Y variant (CC) exhibited an adverse association between BC and total cognition z-score (beta for a doubling in BC concentration: -0.061, 95% CI: -0.115, -0.007), while we did not observe an association in participants with at least one variant genotype (CY or YY) (beta for a doubling in BC concentration: 0.073, 95% CI: -0.081, 0.228; p-value for interaction: 0.11). The pattern of association was similar for analyses considering performance on the Mini-Mental State Examination. There was little evidence to support effect modification of the BC-cognition association by the HFE H63D genotype.

**Conclusions:**

Our data suggest that older adults who lack an HFE C282Y variant may be more susceptible to an adverse effect of traffic-related air pollution exposure on cognition. This finding and the proposed biological mechanism require confirmation.

## Background

While we often focus on mean response to exposures in epidemiology, there are many reasons to be interested in identifying groups of individuals who are particularly susceptible to exposure-related health effects. First, identification of susceptible groups, particularly genetically susceptible groups, may provide insight into the mechanisms behind the association. Epidemiologic gene-environment interaction studies have some advantages over toxicology in this respect because we evaluate susceptibility of people, rather than laboratory animals or cell lines, at doses currently experienced in the population at large [1]. Second, governmental agencies are sometimes charged with or prioritize protecting susceptible or otherwise sensitive groups. Identification and understanding of associations between environmental or occupational exposures and health effects within these groups is essential to understand the true risks associated with exposure in the population and to evaluate the appropriateness of existing or proposed regulatory standards.

Traffic-related air pollution may adversely impact cognition through induction of inflammation and oxidative stress. We previously found a significant association between long-term exposure to black carbon, a marker of traffic-related air pollution, and cognition in older men [2]. Higher levels of black carbon exposure were associated with lower cognitive test performance. Similarly, in a cohort of older German women, living close to a major road, a marker of elevated exposure to traffic-related air pollution, was associated with lower cognitive test scores, especially among those 74 years old and above [3]. Other types of air pollution may also impact cognition through similar mechanisms, as exposure to both coarse and fine particulate matter was associated with faster cognitive decline in a cohort of older women [4] and worse air quality measures were associated with increased disability in activities of daily living and cognitive impairment in elderly Chinese [5]. Finally, there may also be an adverse effect of air pollution exposure on cognition in younger adults or children [6-11].

The hemochromatosis (HFE) gene regulates iron homeostatsis. Two common missense polymorphisms of this gene, HFE C282Y and H63D are associated with the disease state of hemochromatosis, an autosomal recessive genetic disease that causes an increase in absorption of ingested iron. Although the penetrance is low, this can, over time, lead to iron overload, manifesting in higher rates of diabetes, heart disease, and liver disease [12,13]. Variation in these HFE polymorphisms may also appear to impact body burden of other metals, particularly divalent cations like manganese, lead and cadmium [14-18].

Previous studies have reported that HFE polymorphisms modify the association between air pollution exposure and cardiovascular outcomes or risk factors [19-21]. HFE polymorphisms may modify the uptake of metals adhered to particulate matter, altering the inflammatory response to external exposure, which has been proposed as a primary mechanism for the adverse association between air pollution and both cardiovascular endpoints and cognition. For the current study, we hypothesized that the previously observed adverse association between traffic-related air pollution and cognition would be weaker in participants with variant HFE polymorphisms.

## Methods

### Study sample

The current study sample consists of participants from the United States Department of Veterans Affairs (VA) Normative Aging Study (NAS). This ongoing study of men was established in 1963, and since that time participants have been invited to complete an in-person examination every 3 years. At each visit the men provided written informed consent as approved by the VA Boston Healthcare System Institutional Review Board. The current study sample is limited to white participants with complete covariate data who completed at least one cognitive assessment between 1996 and 2007 and for whom we were able to obtain an estimate of long-term exposure to black carbon, a marker of traffic-related air pollution. For this study, we refer to the date of the first cognitive assessment completed during or after 1996 as the baseline visit. As this analysis is a straightforward extension of a previously reported analysis [2] we refer readers to that paper for a more exhaustive description of our methods.

### Exposure assessment

For each participant, we used the average estimated black carbon concentration at their residential address in the year prior to their first cognitive assessment as a measure of long-term exposure to traffic-related air pollution. This estimate was derived using a validated spatio-temporal land use regression model that provides daily estimates of black carbon concentration throughout the greater Boston area starting in 1995 [22]. Estimates of black carbon concentration were log-transformed for use in all analyses.

### Cognitive testing

The content of the battery of cognitive tests used in the NAS has changed since cognitive assessment began in the mid-1990s. We focus on data from 7 cognitive tests administered at most cognitive assessments: the Mini-Mental State Examination (MMSE), the digit span backwards test, a verbal fluency task, constructional praxis, immediate recall of the ten word list, delayed recall of the ten word list, and a pattern comparison task. These seven tests were drawn from established batteries and assess a variety of domains; collectively, we use these tests to provide an assessment of total cognitive function. Most study participants have completed multiple waves of cognitive assessment, all of which were used in our analyses. As the MMSE exhibited a strong ceiling effect, with a large proportion of participants achieving scores at or near the maximum, MMSE scores were dichotomized at ≤25 and were considered in separate analyses (while a score of ≤24 is commonly used as a cutoff for dementia screening in research settings, very few of our participants met this criterion). The 7 scores produced by the remaining 6 cognitive tests (the pattern recognition task produces two unique scores that assess different aspects of cognition), were each standardized to a z-score using the mean and standard deviation of scores from the baseline cognitive assessment in our sample.

### HFE genotyping

For this study we focus on two single nucleotide polymorphisms (SNPs) known to produce missense mutations in HFE: HFE C282Y (rs1800562) and HFE H63D (rs1799945). We genotyped for these SNPs in a subset of NAS participants with archived blood samples using multiplex polymerase chain reaction assays designed with Sequenom SpectroDESIGNER software (Sequenom, Inc., San Diego, CA) by inputting sequences containing the SNP site and 100 bp of flanking sequence on either side. More detailed information on genotyping, including details of laboratory methods, primers, and quality control, can be found elsewhere [20]. Participants for whom valid HFE SNP genotyping could not be obtained were excluded from all analyses. HFE SNP genotypes were coded as a dichotomous variable indicating presence or absence of a variant allele, given the small prevalence of homozygous variant persons in Caucasian populations.

### Statistical methods

As the current dataset excludes participants from the previously reported analyses [2] who lacked valid HFE genotyping, we first reproduced the previously reported associations for the main effects of black carbon exposure on MMSE scores and total cognition in this reduced dataset. We used logistic regression with generalized estimating equations and empirical variance estimates to assess the association between black carbon and low MMSE scores to account for use of multiple waves of cognitive testing per participant. Using this model, we estimated the odds ratio for having a low MMSE score per doubling in black carbon concentrations. We also used a linear mixed model with random intercepts for individual and test to assess the association between black carbon and “total” cognitive function. As with the MMSE analysis, this analysis incorporated multiple waves of cognitive testing per participant for those with more than one assessment. It also accounted for the fact that we have multiple cognitive tests per assessment, as it effectively treats each cognitive test score as a repeat measure of underlying total cognition. Using this model, we estimated the difference in “total cognitive z-score” for a doubling in black carbon concentrations. Due to the small mean number of repeat assessments per person (2.15), we did not investigate the association between black carbon and cognitive trajectories in either the original or the current analysis.

We computed SNP frequencies and assessed Hardy-Weinberg equilibrium within our sample for both HFE SNPs. We began by evaluating the main effect of each of the two HFE SNPs, H63D and C282Y, on the odds of having a low MMSE score and on total cognition in four separate models. We then added multiplicative interaction terms between the pertinent HFE SNP and black carbon to each of these four models to assess the potential for effect modification of the black carbon-cognition association by each HFE SNP. We used Wald tests of the multiplicative interaction term and a p-value threshold of 0.05 to assess whether there was statistically significant support for effect modification of the black carbon-cognition relationship by HFE SNP in each model.

All analyses were adjusted for age at cognitive assessment and the following variables, assessed at baseline: education (≤12, 12-16, 16+ years), alcohol intake (<2/2+ drinks per day), physical activity (<12, 12-30, 30+ metabolic equivalent hours (MET-hrs) per week), diabetes (yes/no), dark fish consumption (less than once a week/once a week or more), computer experience (yes/no), first language (English/not English), percent of the participant’s census tract that is non-white, percent of the adult residents in the participant’s census tract with at least a college degree, an indicator for whether the cognitive data were from the participant’s first cognitive assessment (yes/no), and an indicator for whether the participant was a part-time resident of the greater Boston area (yes/no). In sensitivity analyses, we additionally adjusted all models for hemoglobin, body mass index, smoking status, and estimated long-term exposure to lead (as lead exposure has been associated with cognition in this cohort and the fact that traffic-related air pollution was a major source of lead exposure in the era of leaded gasoline). We used either measured (n = 345) or imputed (n = 270) bone lead concentrations as our estimate of long-term lead exposure, and excluded those without measured tibia lead levels who were missing essential covariates required for imputation (n = 13). Details regarding the measurement or imputation of bone lead concentrations are available elsewhere [2,23,24]. All analyses were performed with R (version 2.10.1; R Development Core Team 2010) or SAS (version 9.2; SAS Institute Inc., Cary, NC).

## Results

Six hundred twenty eight (92%) of the 680 men with data on black carbon exposure, cognition, and all covariates were successfully genotyped for both HFE SNPs. Table 1 summarizes the sample characteristics for the current analysis in total and by HFE SNP genotypes. At baseline, our participants were, on average, 70 years old (SD: 7.1) and had 14.4 years of formal education (SD: 2.6). 540 (86%) of our participants lacked an HFE C282Y variant and 6 (1%) were homozygous for the C282Y variant. 469 (75%) lacked an HFE H63D variant and 18 (3%) were homozygous for the H63D variant. Both SNPs were in Hardy-Weinberg equilibrium (C282Y: *χ*2 = 2.05, p = 0.15; H63D: *χ*2 = 3.32, p = 0.07).

**Table 1:** Baseline characteristics of the cohort (n = 628)

		**HFE C282Y**		**HFE H63D**	
	**All (n = 628)**	**Wild type (n = 540)**	**Variant (n = 88)**	**Wild type (n = 469)**	**Variant (n = 159)**
Variable, mean ± SD					
Age, years	70.0 ± 7.1	70.1 ± 7.1	69.5 ± 7.1	70.0 ± 7.3	70.2 ± 6.3
Education, years	14.4 ± 2.6	14.4 ± 2.7	14.4 ± 2.4	14.5 ± 2.7	14.3 ± 2.5
Black carbon concentration, μg/m^3^	0.58 ± 0.28	0.58 ± 0.28	0.57 ± 0.27	0.59 ± 0.29	0.56 ± 0.24
Body mass index, kg/m^2^	27.9 ± 3.9	27.9 ± 4.0	28.0 ± 3.7	28.0 ± 4.1	27.6 ± 3.5
Physical activity, MET-hr/week	16.6 ± 20.1	16.3 ± 19.9	18.0 ± 21.8	16.7 ± 20.6	16.3 ± 18.8
% of census tract: adults with a college degree	39.9 ± 16.5	40.3 ± 16.8	37.1 ± 14.5	40.2 ± 16.5	39.1 ± 16.8
% of census tract: non-white	10.9 ± 11.4	10.9 ± 11.1	10.4 ± 13.0	10.4 ± 10.6	12.3 ± 13.4
Variable, n (%)					
Diabetic	94 (15.0)	79 (14.6)	15 (17.0)	69 (14.7)	25 (15.7)
Hypertensive	415 (66.1)	367 (68.0)	48 (54.5)	304 (64.8)	111 (69.8)
Smoking					
Never	185 (29.5)	161 (29.8)	24 (27.3)	140 (29.9)	45 (28.3)
Former	410 (65.3)	351 (65.0)	59 (67.0)	302 (64.4)	108 (67.9)
Current	33 (5.3)	28 (5.2)	5 (5.7)	27 (5.8)	6 (3.8)
Consume ≥2 alcoholic drinks per day	157 (25.0)	137 (25.4)	20 (22.7)	122 (26.0)	35 (22.0)
Consume dark fish ≥1/week	94 (15.0)	81 (15.0)	13 (14.8)	68 (14.5)	26 (16.4)
English as first language	538 (85.7)	454 (84.1)	84 (95.5)	400 (85.3)	138 (86.8)
Has computer experience	255 (40.6)	218 (40.4)	37 (42.0)	193 (41.2)	62 (39.0)

Abbreviations: *HFE*, hemochromatosis gene; *SD*, standard deviation; *MET-hr/week*, metabolic equivalent hours per week.

In the current study sample, the direction and magnitude of the association between black carbon and cognitive function in fully adjusted models was materially unchanged compared to previously reported analyses (Additional file 1: Table S1). As expected, there was little evidence to support a main effect of either HFE variant on cognition. The presence of an HFE C282Y variant did not appear to be associated with total cognition (beta: -0.063, 95% CI: -0.142, 0.017) or MMSE performance (odds ratio (OR): 0.92, 95% CI: 0.64, 1.35). Similarly, presence of an HFE H63D variant was not associated with either total cognition (beta: 0.022, 95% CI: -0.041, 0.085) or MMSE performance (OR: 0.98, 95% CI: 0.75, 1.28).

Consideration of regression models with interaction terms between HFE genotype and black carbon suggests that HFE C282Y, but not HFE H63D, modifies the association between black carbon concentrations and total cognitive function (Table 2). Although the p-values for interaction did not achieve the threshold for statistical significance in any model, the adverse association between black carbon exposure and cognition appears to be exclusive to those lacking the HFE C282Y variant. While participants who lacked an HFE C282Y variant exhibited strong adverse associations between BC and total cognition, a magnitude of effect that is equivalent to approximately 2.1 years of age in our data, we did not observe an adverse association for participants with at least one variant genotype. Similarly, we found elevated odds of low MMSE scores for a doubling in BC concentrations for participants who lacked an HFE C282Y variant but little evidence to support an association in participants with at least one copy of the HFE C282Y variant (Table 3).

**Table 2:** The association between a doubling in BC concentration and total cognitive z-score by HFE polymorphisms

	**Expected difference in total cognitive z-score**^**a**^**(95% confidence interval)**	**p-value for interaction**
HFE C282Y		
Wild Type	−0.061 (-0.115, -0.007)	0.11
Variant	0.073 (-0.081, 0.228)	
HFE H63D		
Wild Type	−0.071 (-0.132, -0.009)	0.64
Variant	−0.024 (-0.131, 0.082)	

Abbreviations: *HFE*, hemochromatosis gene; *BC*, black carbon.

^a^Adjusted for age, education, first language, computer experience, physical activity, alcohol consumption, diabetes, dark fish consumption, percentage of residential census tract that is nonwhite, percentage of residential census tract adults with a college degree, indicator for first cognitive assessment, and indicator for part-time resident.

**Table 3:** The association between a doubling in BC concentration and low MMSE scores by HFE polymorphisms

	**Odds ratio**^**a**^**(95% confidence interval)**	**p-value for interaction**
HFE C282Y		
Wild Type	1.37 (1.08, 1.73)	0.20
Variant	0.91 (0.50, 1.64)	
HFE H63D		
Wild Type	1.24 (0.97, 1.59)	0.22
Variant	1.74 (1.06, 2.87)	

Abbreviations: *HFE*, hemochromatosis gene; *MMSE*, Mini-Mental State Examination; *BC*, black carbon.

^a^Adjusted for age, education, first language, computer experience, physical activity, alcohol consumption, diabetes, dark fish consumption, percentage of residential census tract that is nonwhite, percentage of residential census tract adults with a college degree, indicator for first cognitive assessment, and indicator for part-time resident.

We found little support for modification of the black carbon-cognition association by HFE H63D for either total cognition or MMSE performance. Although we observed statistically significant associations between black carbon and cognition within groups defined by HFE H63D genotype, the p-values for interaction did not achieve statistical significance in either model, and the pattern of association across those with and without the HFE H63D variant differed by outcome. In analyses of total cognition, we observed a statistically significant adverse association between black carbon concentrations among HFE H63D homozygous wild type participants, but not among those possessing at least one variant allele (Table 2). However, we observed the opposite pattern in analyses considering MMSE scores (Table 3), where we found a statistically significant adverse association in those with at least one H63D variant, but not in those who were homozygous wild type.

In sensitivity analyses, additional adjustment for hemoglobin, body mass index, smoking, or past lead exposure did not appreciably change effect estimates (data not shown).

## Discussion

Although the statistical evidence to support the claim that the HFE C282Y polymorphism modifies the association between traffic-related air pollution and cognitive function in our sample of older men is weak, the pattern of response across groups is intriguing and suggests the presence of effect modification by HFE C282Y. Participants possessing a variant HFE C282Y polymorphism appear to be protected from the adverse association between black carbon and cognition, while participants who lacked the C282Y variant exhibit strong adverse associations. We found little support for modification of the association between traffic-related air pollution and cognition by HFE H63D.

This is the first study to investigate effect modification by HFE of the air pollution-cognition relationship. These findings are in line with reported effect modification by HFE of the relationship between exposure to air pollution and cardiovascular outcomes or risk factors in the NAS cohort. Persons lacking HFE variant genotypes exhibit strong adverse associations between exposure to fine particulate matter (PM) and heart rate variability, while there is little evidence for an association among those possessing an HFE variant, and this pattern was driven primarily by the HFE C282Y SNP [20]. Similarly, higher exposures to black carbon or PM2.5 were associated with higher plasma homocysteine concentrations in participants who lacked the HFE C282Y variant, but no association was observed in participants possessing at least one HFE C282Y variant [19]. A similar pattern was observed for modification by HFE C282Y of the association between traffic-related air pollution and heart-rate-corrected (QT) interval, an electrocardiographic marker of ventricular repolarization that is associated with arrhythmia and cardiac death [21].

The mechanism by which exposure to traffic-related air pollution may contribute to cognitive impairment is unknown, but likely involves inhalation of particulates. In animal experiments, exposure to fine or ultrafine particulate matter appears to induce inflammation, lipid peroxidation, and neuronal degeneration in the central nervous system [25-28]. Metals, including iron, are often found adsorbed onto the surface of traffic-related particulates [29] and exposure to metals is associated with induction of oxidative stress and inflammation [30]. Oxidative stress and inflammation may contribute to the development of cognitive impairment directly or through inducement of cardiovascular problems.

HFE modulates intracellular uptake of iron and other metals. Variants of HFE are associated with the disease hemochromatosis, which is characterized by iron overload due to excessive uptake of iron from the gastrointestinal tract [12]. Studies of HFE modulation of the health effects of air pollution, including the current study, generally find that variants in HFE protect against the adverse impact of air pollution exposure [19,20]. These findings may be attributable to HFE modulation of uptake of metals from the lung. Exposure to traffic-related air pollution occurs primarily through inhalation. In order for traffic-related air pollution to adversely impact human health, some component of traffic-related air pollution must enter the body, either through penetration of the respiratory epithelium, ingestion, or through translocation up the olfactory nerve. Variants of HFE C282Y and H63D are associated with increased iron uptake from the gut, and higher body stores of iron may down-regulate overall metal absorption, including absorption from the lung of metals adhered to inhaled air pollution. In animal models, rats fed a high-iron diet or exposed to iron oxide exhibited less absorption of manganese after intratracheal installation compared to those fed a regular diet [17,18]. This reduction in pulmonary absorption was paralleled by lower uptake by other organs, including the brain [18]. Therefore, HFE genotype, associated iron levels, and iron homeostatic mechanisms could influence the toxicity of exposure to traffic-related air pollution through modulation of metal uptake. Lower uptake of adsorbed metals would be expected to reduce the associated systemic and brain-based oxidative stress and inflammation and lessen any adverse effect of air pollution on cognition. This mechanistic hypothesis might explain why we did not see an adverse association between air pollution and cognition in carriers of the HFE C282Y variant.

We also note that the evidence to support effect modification of the association between air pollution and cognition was stronger when we considered the C282Y variant than when we considered the H63D variant, which is consistent with the relative degree of function of these SNPs. The HFE protein produced by the C282Y variant, but not the H63D variant protein, is often degraded before final processing and is not expressed on the cell surface [31] and does not associate with transferrin [32]. As such, the C282Y variant is associated with greater loss of function compared to the H63D variant, resulting in increased iron transfer in the gut. In this case, we may expect lower respiratory intake of adsorbed metals, and therefore lower levels of air pollution-related oxidative stress and inflammation, in those with the C282Y variant than in those with the H63D variant.

We acknowledge that our study has several limitations. Use of BC exposure estimates based on residential address may misclassify long-term exposure. However, given the age and residential stability of our cohort, misclassification of our exposure due to occupation, commuting, or relocation is expected to be minimal and largely unrelated to cognition or HFE status, suggesting bias towards the null. Non-differential misclassification of cognitive status is likely, but is mitigated by the use of all available cognitive data to consider the association between BC and total metrics of cognition. It is possible that residual confounding may bias our results. However, we were able to adjust for many known predictors of cognition, including several measures of socioeconomic status. Furthermore, we expect confounding by other classes of air pollution to be minimal, as the correlation between traffic-related air pollution and regional pollutants is relatively low due to differences in the spatial-temporal distribution of these types of pollutants. Selection bias is a concern, but because both poor cognition and exposure to traffic-related air pollution predict morbidity and mortality [33-36], we would expect selection bias to mask the expected adverse association or create a false protective association. While this may contribute to the absence of an adverse association among participants with HFE C282Y variants, it would not account for the strong adverse association observed within those who lacked HFE C282Y variants. While we are unable to concretely attribute our findings to a particular aspect of traffic-related exposure, modification of the BC-cognition association by HFE implicates metals adhered to particulate matter as a likely toxic agent. While population substructure may induce spurious associations in gene-environment interaction studies, this is unlikely to account for our findings given our homogeneous study sample of white men. Finally, our sample size and the number of participants with the variant HFE alleles are small, which makes it difficult to detect a subtle effect and increases the possibility that our findings are due to chance. This analysis requires replication in an independent sample.

## Conclusions

In conclusion, our study suggests that persons who lack a HFE C282Y variant polymorphism may be more susceptible to adverse effects of exposure to traffic-related air pollution on cognition than those possessing a variant. The mechanism by which HFE modifies this association may involve metal transport and inflammation pathways. As with any candidate gene-environment interaction study, this analysis requires independent confirmation.

## Abbreviations

BC: Black carbon; BMI: Body mass index; HFE: Hemochromatosis gene; CI: Confidence interval; MET-hrs: Metabolic equivalent hours; MMSE: Mini-Mental State Examination; NAS: Normative Aging Study; OR: Odds ratio; QT interval: Heart-rate-corrected interval; VA: United States Department of Veterans Affairs.

## Competing interests

The authors declare they have no competing interests.

## Authors’ contributions

MCP made substantial contributions to the study conception and design, analysis and interpretation of the data, and drafted the manuscript. MGW and JS made substantial contributions to the study conception and design, analysis and interpretation of the data, and revised the manuscript critically for important intellectual content. BAC, SEA, AS made substantial contributions to the acquisition of data and revised the manuscript critically for important intellectual content. ROW made substantial contributions to the analysis or interpretation of the data and revised the manuscript critically for important intellectual content. All authors have given final approval of the version to be published.

## Supplementary Material

Additional file 1: Table S1 Comparison the association between a doubling in BC concentration and cognition in the HFE dataset to the previously reported association in the full dataset. Table showing the main effect of BC exposure in the full dataset used in the original analyses and the reduced dataset used in the current analyses. Click here for file
