# Supplementary material for: Modification by hemochromatosis gene polymorphisms of the association between traffic-related air pollution and cognition in older men: a cohort study
Source: Environ Health. 2013 Feb 15;12:16. doi: 10.1186/1476-069X-12-16 (PMC3599892; doi:10.1186/1476-069X-12-16)
Supplement: Additional file 1: Table S1 — Comparison the association between a doubling in BC concentration and cognition in the HFE dataset to the previously reported association in the full dataset. Table showing the main effect of BC exposure in the full dataset used in the original analyses and the reduced dataset used in the current analyses. [file 1476-069X-12-16-S1.pdf]

Additional File Table 1. Comparison the association between a doubling in BC concentration and cognition in the HFE dataset to the previously reported association in the full dataset

		Association Type	Effect Estimate[a] (95% confidence interval)
**Full dataset (n=680)**			
	Total Cognitive Function	beta	-0.054 (-0.103, -0.006)
	MMSE	OR	1.3 (1.1, 1.6)
**HFE dataset (n=628)**			
	Total Cognitive Function	beta	-0.047 (-0.098, 0.005)
	MMSE	OR	1.3 (1.1, 1.6)

Abbreviations: HFE, hemochromatosis gene; OR, odds ratio

[a]Adjusted for age, education, first language, computer experience, physical activity, alcohol consumption, diabetes, dark fish consumption, percentage of residential census tract that is nonwhite, percentage of residential census tract adults with a college degree, indicator for first cognitive assessment, and indicator for part-time resident.
